# Evaluating Umbilical Masses: Lessons Learned From Three Elderly Patients

**DOI:** 10.7759/cureus.67667

**Published:** 2024-08-24

**Authors:** Tze Hui Soo, Marylyn Ganapragasam, Subapriya Suppiah, Norafida Bahari

**Affiliations:** 1 Department of Radiology, Faculty of Medicine and Health Sciences, Universiti Putra Malaysia, Serdang, MYS

**Keywords:** sister mary joseph nodule, umbilical nodule, umbilical lesion, infected urachal cyst, primary umbilical leiomyosarcoma

## Abstract

The umbilicus, an essential embryonic organ, connects the foetus to the placenta. Postnatally, its remnants can lead to both benign and malignant lesions. Tumour metastasis to the umbilicus, though rare, poses significant diagnostic and therapeutic challenges due to overlapping clinical and radiological features with benign conditions. In this case series, we present three cases of elderly women with similar presentations of umbilical or infraumbilical lesions, investigated using standard imaging protocols, which subsequently led to different diagnoses and management plans. Case 1 involves a 64-year-old postmenopausal woman, who presented with an enlarging umbilical mass and serous discharge. Imaging revealed a vascularized lesion in the umbilicus and a suspicious adnexal mass, which was confirmed to be a Sister Mary Joseph nodule secondary to high-grade serous carcinoma of the right ovary. Case 2 involves an 80-year-old diabetic woman, who presented with a painful umbilical mass. Imaging revealed an aggressive-looking umbilical lesion, which was confirmed to be liposarcoma. Case 3 involves an 80-year-old woman, who presented with infraumbilical abdominal swelling and fever. Imaging revealed an infected urachal cyst. Histopathology confirmed an abscess associated with *Actinomycetes* infection. An umbilical lesion in an adult, particularly an elderly patient, that does not respond to typical treatment should raise the suspicion of a more sinister diagnosis. Integration of clinical, radiological, and pathological data is crucial for accurate diagnosis and effective management.

## Introduction

The umbilicus is an important embryonic organ that connects the developing foetus and the placenta during foetal development. At week four of embryo development, the umbilical cord consists of two umbilical arteries, one umbilical vein, an allantoic duct, and a vitelline duct. After delivery, the umbilical cord stump heals into a scar and can sometimes retain epithelial tissue [1]. The umbilical artery obliterates and becomes the medial umbilical ligament. The obliterated umbilical vein produces ligamentum teres (also known as round ligament) between the anterior surface of the liver and the anterior abdominal wall [2]. The urachus is a fibrous remnant of the allantois, which originally connected the foetal urinary bladder to the umbilicus, forming the median umbilical ligament in adults. The vitelline duct, also known as the omphalomesenteric duct, is a structure in the developing embryo that connects the yolk sac to the midgut lumen. Typically, this duct obliterates and disappears during foetal development, forming no significant structure in normal adult anatomy. However, if the obliteration process is incomplete or abnormal, remnants of the vitelline duct can persist and lead to the most common Meckel's diverticulum. Due to this complex anatomical connection of the umbilical remnant with the abdominopelvic cavity, it is not surprising that the umbilicus is an occasional site for tumour metastasis, albeit rare [3]. Other possible routes of metastasis to the umbilicus may include arterial, venous, and lymphatic channels; however, the direct extension of the tumour through the peritoneum appears to be the most favourable pathway.

Tumour emboli can enter the systemic circulation and be delivered to the umbilicus via the rich arterial network, particularly the superior and inferior epigastric arteries, which are continuations of the internal mammary and external iliac arteries, respectively [4]. The paraumbilical veins that connect to the portal system play a significant role in venous metastasis from primary intra-abdominal malignancies. Lymphatic spread is another primary pathway for umbilical metastasis. The umbilicus is connected to a rich network of lymphatic vessels that drain the abdominal and pelvic regions. The lymphatic vessels that drain into the para-aortic, external iliac, and inguinal lymph nodes provide a pathway for cancer cells to travel to the umbilicus [5]. The pathophysiological mechanisms underlying these routes involve complex interactions between tumour cells and the endothelial cells lining blood and lymphatic vessels. Tumour cells can induce angiogenesis, which facilitates their growth and dissemination. Angiogenesis is driven by factors such as vascular endothelial growth factor and basic fibroblast growth factor, which promote the proliferation and migration of endothelial cells [6]. Additionally, the lymphangiogenesis process, which involves the formation of new lymphatic vessels, is critical for lymphatic spread and is often upregulated in cancers with high metastatic potential. The overlapping clinical and radiological features of benign and malignant umbilical nodules cannot be over-stressed, posing diagnostic challenges that are imperative to address, as the management strategies differ significantly. Benign lesions may be treated conservatively or with minor surgical intervention, while malignant lesion necessitates a thorough oncological workup to identify and address the primary malignancy. This case series describes three adult cases with umbilical lesions with varied diagnosis, management, and a brief radiological approach for adult umbilical lesions.

## Case presentation

Case 1

**Figure 1:**
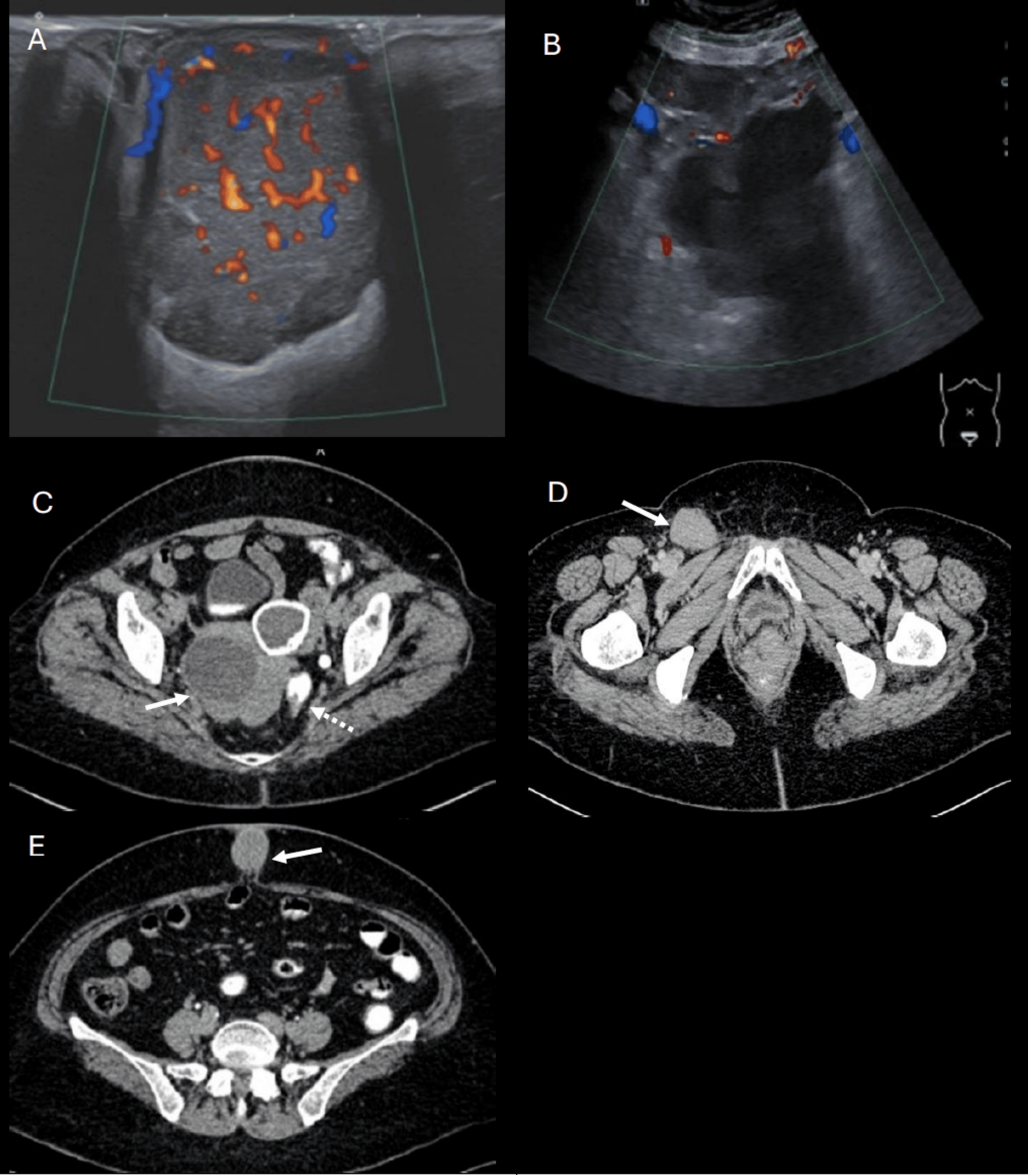
Ultrasound of the umbilicus (A) and abdomen (B) revealed a well-defined, vascularized, heterogeneous hypoechoic lesion within the umbilicus and a suspicious adnexal mass, respectively. Contrast-enhanced CT of the abdomen (axial view) confirmed the presence of a right complex adnexal mass (arrow, C), displacing the adjacent rectal and sigmoid colon (dash arrow, C) with poor fat demarcation. The enlarged right inguinal lymph node, measuring 2.4 x 3.6 cm, showed loss of normal fatty hilum (arrow, D). An enhancing soft tissue umbilical lesion (arrow, E) indicative of metastatic carcinoma was also evident.

A 64-year-old multiparous, postmenopausal woman presented with a two-month history of an enlarging umbilical mass accompanied by foul-smelling serous discharge and pruritus. The patient reported no gastrointestinal symptoms or family history of malignancy. Initially diagnosed with an umbilical abscess but the mass did not improve despite antibiotic therapy. Physical examination revealed a hard, fixed, red-purplish umbilical mass measuring 3.0 x 3.0 cm. A swab culture grew *Pseudomonas aeruginosa*, prompting treatment with intravenous augmentin. Ultrasound (USG) of the abdomen identified a well-defined, vascularized, heterogeneous hypoechoic lesion within the umbilicus, measuring 2.5 x 2.1 x 2.8 cm (Figure 1A). Additionally, a suspicious-looking lobulated hypoechoic mass seen in the right adnexa, measuring 0.9 x 10.2 x 12.2 cm, was noted (Figure 1B). Subsequent contrast-enhanced computed tomography (CECT) of the abdomen confirmed the right complex adnexal mass (Figure 1C), locoregional mass effect, and right inguinal lymphadenopathy (Figure 1D). Again, the enhancing soft tissue umbilical lesion (Figure 1E) was seen and this raised the suspicion of a Sister Mary Joseph nodule.

Serum CA125 was elevated at 514 U/ml. Upon further questioning, the patient reported occasional postmenopausal bleeding over the past year. A wedge resection of the umbilical mass and fine-needle aspiration cytology (FNAC) of the right inguinal lymph node confirmed metastatic carcinoma of likely gynaecological origin. She completed six cycles of neoadjuvant chemotherapy, followed by debulking surgery, including total abdominal hysterectomy, bilateral salpingo-oophorectomy, omentectomy, pelvic lymph node dissection, and umbilical resection. Histopathological examination (HPE) confirmed high-grade serous carcinoma in the right tubo-ovarian mass. Postoperatively, her CA125 level dropped to 5 U/ml, and she remained well throughout the follow-up. A follow-up staging CT revealed no evidence of local recurrence, and she is now under regular surveillance by her gynaecologist.

Case 2

**Figure 2:**
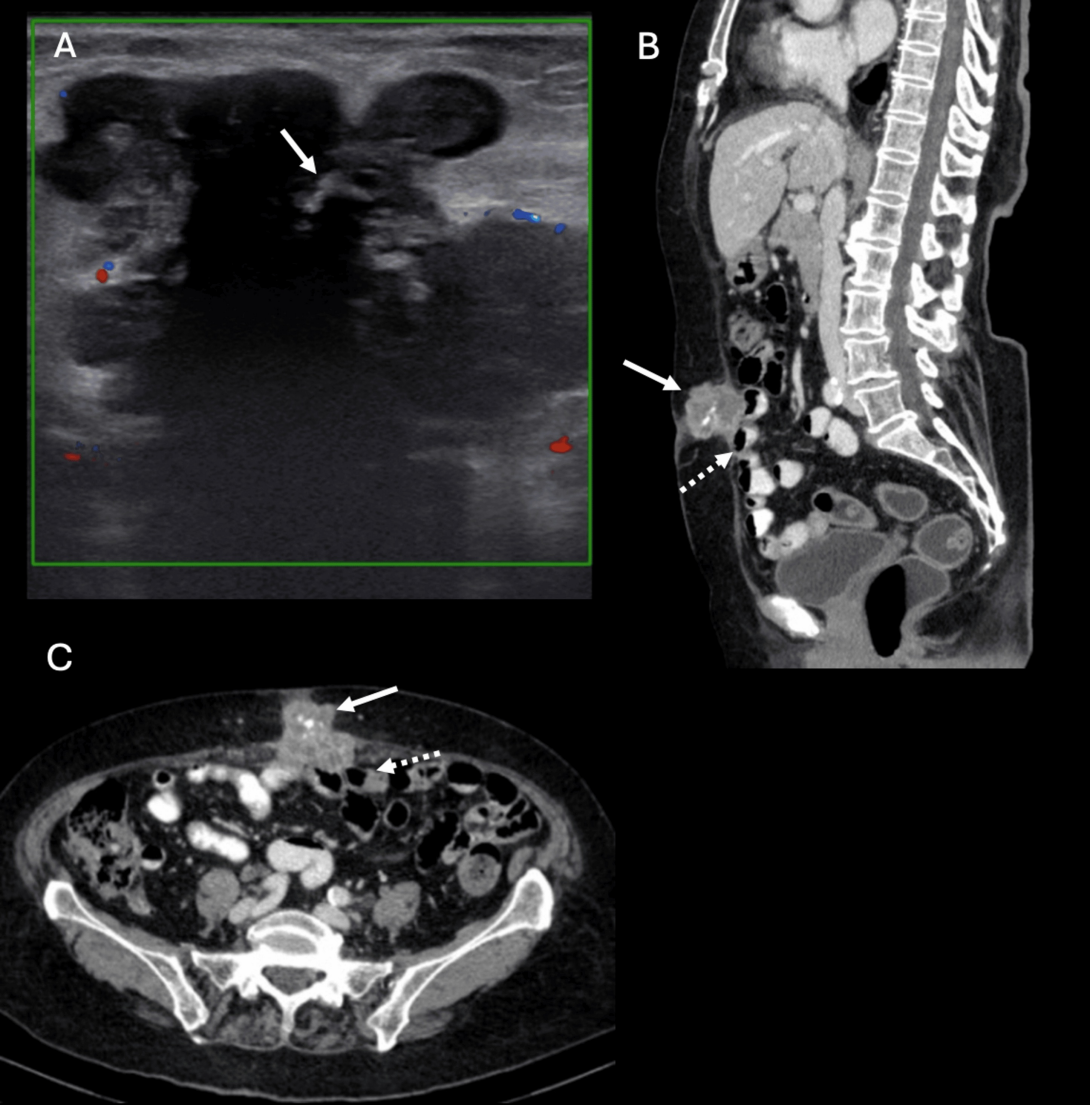
Ultrasound of the umbilicus (A) shows a well-defined, lobulated, hypoechoic umbilical lesion with internal calcifications (arrow, A), exhibiting minimal peripheral vascularity. Axial (arrow, B) and sagittal (arrow, C) views of contrast-enhanced CT of the abdomen further delineate the lesion's extension into the subcutaneous fat and preperitoneal space, compromising the fat plane with the adjacent ileum (dash arrow in image B and C).

An 80-year-old woman with underlying diabetes and hypertension presented with umbilical swelling for three to four months, accompanied by pain. Physical examination revealed a palpable, hard, fixed mass in the umbilicus, measuring approximately 5.0 x 5.0 cm. An abdominal ultrasound identified a well-defined, lobulated, hypoechoic lesion with internal calcifications, measuring 5.0 x 3.6 x 3.8 cm and showing minimal peripheral vascularity (Figure 2A). CECT of the abdomen further characterized the umbilical mass with extension into the subcutaneous fat and preperitoneal space, compromising the fat plane with the adjacent ileum (Figures 2B, 2C).

HPE suggested a mesenchymal tumour with smooth muscle differentiation, confirmed by vimentin and smooth muscle actin (SMA) positivity, indicative of liposarcomatous differentiation. She underwent wide local excision of the umbilical mass, small bowel resection, and end-to-end stapler anastomosis. HPE of the resected small bowel did not show visceral spread. Given the patient's advanced age, a palliative approach was adopted, and she was referred to the oncology team for regular monitoring.

Case 3

**Figure 3:**
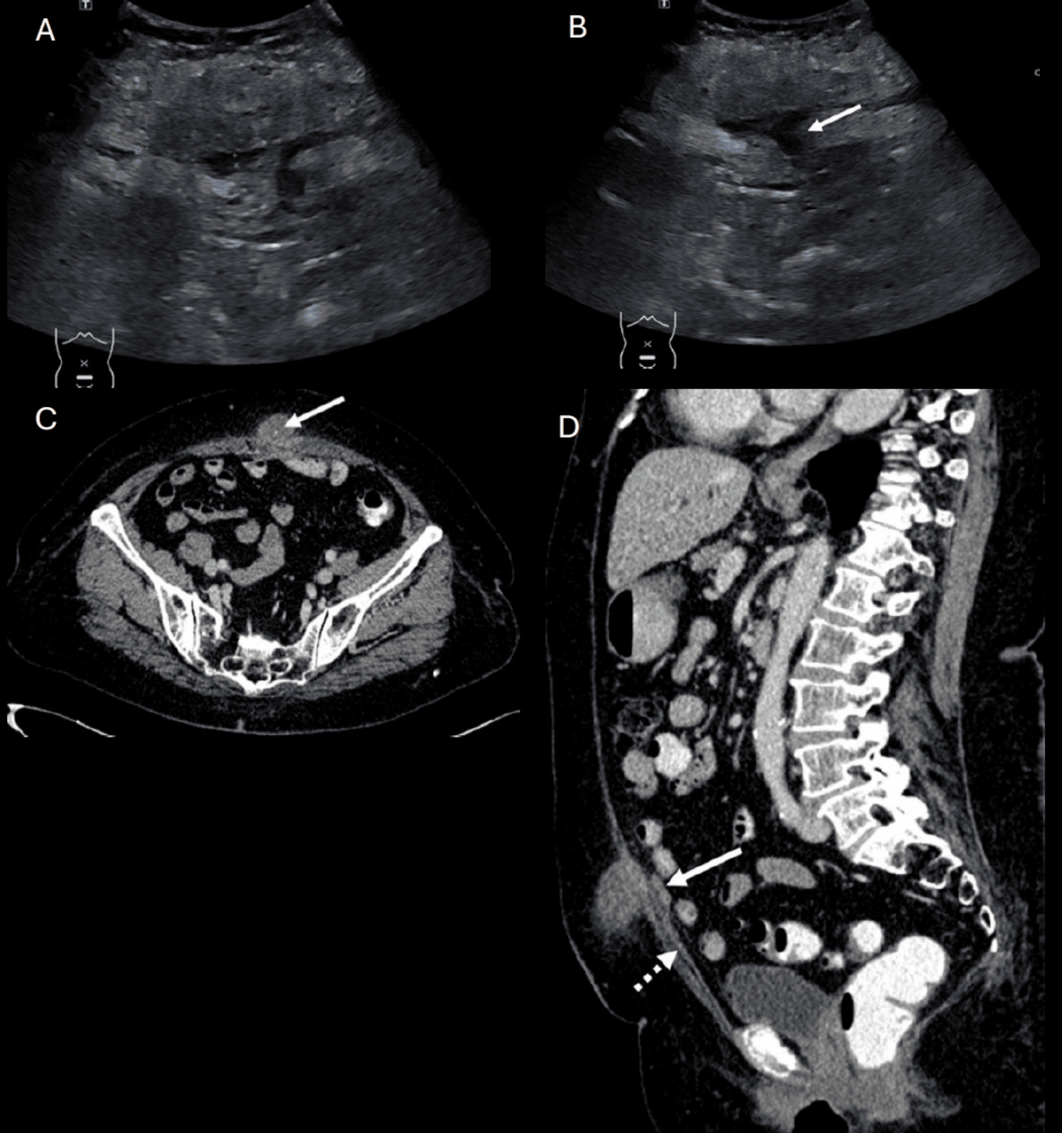
Ultrasound of the anterior abdominal wall (A and B) reveals an avascular, poorly defined, and heterogeneous echogenic subcutaneous collection in the lower anterior abdomen, measuring 2.6 x 5.9 x 4.6 cm (AP x W x CC), with trace amounts of liquefied components (arrow, B). The overlying subcutaneous tissue is notably thickened and oedematous. Subsequent axial contrast-enhanced CT of the abdomen (C) corroborates the ultrasonographic findings, further elucidating the heterogeneous subcutaneous lesion and identifying a small calcification focus within it (arrow, C), along with subcutaneous fat stranding. Additionally, the sagittal contrast-enhanced CT view (D) depicts the thickened proximal attachment of the medial umbilical ligament (dash arrow, D) and urachal remnant (arrow, D), providing further characterization of the abnormality. AP = anteroposterior; W = width; CC = craniocaudal.

An 80-year-old woman with a history of dyslipidaemia presented to the outpatient surgical clinic with a one-month history of lower abdominal swelling and a one-week history of fever. Despite completing a course of antibiotics, her symptoms showed no notable improvement. The patient denied any lower urinary tract symptoms, umbilical pain, or discharge. On physical examination, her abdomen revealed a firm, erythematous midline lower abdominal swelling measuring 5 x 5 cm with ill-defined edges and no signs of peritonitis. Due to the persistence of symptoms and lack of clinical improvement, she was admitted for further investigation and administration of intravenous antibiotics. Laboratory findings indicated normal C-reactive protein levels and blood cultures did not isolate any pathogens. USG of the abdomen demonstrated an avascular, ill-defined, heterogeneous echogenic subcutaneous lesion in the anterior lower abdomen, measuring 2.6 x 5.9 x 4.6 cm (anteroposterior x width x craniocaudal), with trace amounts of liquefied components (Figures 3A, 3B). The overlying subcutaneous tissue was thickened and oedematous. These USG findings were further elucidated by subsequent CECT of the abdomen, which revealed a tiny calcification focus within the lesion with an average of 41 HU (Figure 3C). The mass was abutting the underlying left rectus abdominis muscle, which showed mild enhancement and thickening. Additionally, the proximal attachment of the medial umbilical ligament and urachal remnant was thickened (Figure 3D).

No other collections were observed in the peritoneal organs, and there was no omental caking, suspicious malignancy, significant lymphadenopathy, or ascites. Given the findings, excision of the urachal cyst was planned once the infection was under control. Intraoperatively, the infected cyst was identified within the subcutaneous layer, exhibiting a blunt end without extension into the intraperitoneal cavity or rectus muscle. The surrounding tissue was inflamed, with minimal purulent discharge observed during manipulation. No urine was detected. The urachal cyst was excised, and the wound was irrigated with a copious amount of povidone-iodine solution. Histopathological examination confirmed the presence of an abscess associated with* Actinomycetes* infection. Postoperatively, the patient recovered well, with no subsequent complications reported.

## Discussion

Umbilical lesions are rare and can be benign or malignant in nature. Sister Mary Joseph nodule (SMJN) is an uncommon palpable umbilical metastasis commonly seen in women. It was first described in 1949 by Sir Hamilton Bailey in honour of Sister Mary Joseph, who was the first to identify the relationship between umbilical nodules and intra-abdominal malignancy [7]. The incidence ranges from 1% to 9% in autopsy and represents only 10% of skin metastasis. Origin from gastrointestinal (stomach, colorectal, and pancreas) and genitourinary (ovaries, endometrium, cervix, and prostate) systems constitute the majority of the primary tumour. Of these, the most common histological type is adenocarcinoma. In some cases, the primary source remains unknown. Despite its historical recognition, the clinical implications of an SMJN remain relevant, warranting attention to detail in physical examinations [8]. Common presentation is an irregular margin, painful, hard lump usually ranging from 0.5 to 2 cm. There may be umbilical discharge of either blood, serous, purulent, or mucus. At times, ulceration with superimposed infection can be seen and may be the only and first presentation (20%) of an occult underlying malignancy, as observed in our case 1. Its unspectacular clinical appearance, supported by findings from swab culture that yielded *Pseudomonas* species, led to the misdiagnosis of umbilical abscess in the initial phase. It may also represent disease recurrence in known malignancy. Differentiating between benign umbilical tumours and SJMN is critical, as the latter typically indicates metastatic disease and carries a grave prognosis [9]. Clinically, benign umbilical nodules such as umbilical hernias, granulomas, and inclusion cysts typically present as soft, painless, and slow-growing masses. These benign nodules may be associated with local symptoms but rarely present systemic signs of malignancy. For instance, umbilical hernias are often reducible and may cause discomfort when incarcerated or strangulated. In contrast, SMJNs are usually firm, non-tender, and often accompanied by systemic symptoms like weight loss, anorexia, and fatigue [10]. Radiologically, benign nodules often appear as well-defined, homogeneous, and cystic structures in ultrasound, whereas SMJNs tend to show irregular, solid, and heterogeneous patterns [9]. CT and MRI scans provide detailed insights; benign lesions typically lack aggressive features such as invasion into surrounding tissues, while SMJNs often demonstrate irregular borders and evidence of metastatic spread, including lymphadenopathy and involvement of adjacent organs [11]. Fine needle biopsy plays an important role in confirming malignancy in a timely manner to improve patient survival. Although it can be easily overlooked, this nodule provides valuable diagnostic information and serves as an ominous sign regarding prognosis, necessitating prompt investigation, and comprehensive evaluation to identify and treat the primary malignancy.

Primary neoplasm of the umbilical is extremely rare. Using PubMed as a search engine with the keyword “primary umbilical cancer/carcinoma/malignancy/sarcoma” revealed a paucity of cases worldwide from 1946 up to date. Fewer than 10 cases of primary umbilical tumour, which consist of melanoma, basal cell carcinoma, squamous cell carcinoma, and primary umbilical adenocarcinoma, have been documented. Umbilical leiomyosarcoma has never been reported thus far and our case 2 serves as the first case reported in the literature. Liposarcoma is a malignant tumour originating from adipose tissue. It is among the most common types of soft tissue sarcomas and predominantly affects adults, particularly those between the ages of 40 and 60 years [12]. This tumour can arise in any location where fat is present, but it most frequently occurs in the extremities, particularly the thigh, accounting for approximately 50% of cases, and in the retroperitoneum, which accounts for about 20% of cases [12]. Less commonly, liposarcomas may be found in the shoulder, buttock, and, rarely, in the mediastinum or other visceral sites. The prevalence of liposarcoma varies among its subtypes, with well-differentiated liposarcoma being the most common and pleomorphic liposarcoma the least common [12]. MRI and CT are the primary imaging modalities used. Well-differentiated liposarcomas typically present as large, well-circumscribed masses with high fat content, appearing hyperintense on T1-weighted images (T1WI) and hypointense on T2-weighted images (T2WI), with thin septa and no significant enhancement after gadolinium administration [13]. Dedifferentiated liposarcomas, on the other hand, often show a heterogeneous appearance with both fatty and non-fatty components, reflecting areas of high cellularity and necrosis. Myxoid liposarcomas display a distinctive “pseudocystic” appearance due to the presence of myxoid stroma, appearing hyperintense on T2WI and hypointense on T1WI, often with a characteristic “ring and arc” pattern of enhancement post-gadolinium administration [14]. CT scans are particularly useful in detecting retroperitoneal liposarcomas, providing detailed information about the tumour’s relationship with adjacent structures and any evidence of metastasis. PET-CT may be employed to evaluate the metabolic activity of the tumour and assess for distant spread, particularly in high-grade subtypes [15]. Although imaging modalities provide detailed anatomical information and are essential for planning the surgical approach, histopathological examination is the cornerstone for the definitive diagnosis. Umbilical liposarcoma provides no exception to this guideline.

Although more than 50% of umbilical nodules are often associated with a malignant process, their benign entity cannot be overlooked, especially in younger populations. Benign causes include papilloma, epithelial inclusion cyst, lipoma, neurofibroma, fibroma, and endometriosis. Incomplete obliteration of the allantois leads to four clinically significant anomalies, namely, patent urachus, urachal cyst, urachal sinus, and urachal diverticulum. Infected urachal cysts present a small fraction of all urachal anomalies, which themselves are uncommon, with an incidence of about one in 5,000 live births [16]. The majority of urachal anomalies are diagnosed in children, but infected urachal cysts can present at any age, with a notable number of cases occurring in adults due to delayed diagnosis or sudden onset of symptoms. It presents a unique clinical challenge due to their potential complications and varied symptomatology. These cysts can lead to severe infections such as peritonitis when ruptured intraperitoneally. The infection in urachal cysts can stem from various sources, including hematogenous or lymphatic routes, as well as direct spread from adjacent structures like the bladder or umbilicus. Patients with infected urachal cysts commonly exhibit symptoms such as abdominal pain, fever, umbilical discharge, and the presence of a midline mass [17]. Infected cysts often display specific features like increased echogenicity on ultrasound and thick-walled cystic or mixed attenuation on CT scans [18]. Diagnosing infected urachal cysts is not straightforward in some cases, as there are a wide range of conditions that can present with similar imaging features. Of these, appendicitis, particularly when located near the midline, can present with peri-appendiceal abscesses showing fluid collections and inflammation. Omphalitis, infected umbilical hernia, and infected inclusion cysts can further complicate the diagnostic journey. The definitive treatment for infected urachal cysts typically involves a combination of intravenous antibiotics to control the infection, followed by complete excision of the urachal remnant.

The radiological approach to diagnosing and differentiating umbilical nodules involves various imaging modalities tailored to the suspected pathology. High-frequency linear ultrasound transducers are the ideal first-choice imaging modality regardless of the suspected pathology. The primary purpose of ultrasound in an umbilical lesion is to confirm that it is actually a discrete mass rather than an abdominal wall hernia, which is more commonly encountered in clinical practice. Once a discrete lesion is identified, its fat, fluid, or solid composition can be determined, which guides the radiologist to different imaging algorithms and differential diagnoses [19]. The differential diagnosis for fat-containing lesions encompasses lipoma, liposarcoma, and vascular malformation. For cystic masses, common diagnostic considerations include seromas, hematomas, and abscesses. Solid masses, on the other hand, may indicate conditions such as endometriosis, desmoid tumours, fibromatous tumours, peripheral nerve sheath tumours, or metastatic lesions [19]. CT scans offer detailed cross-sectional images that are instrumental in identifying the extent of lesions and their relation to surrounding structures, while MRI provides superior soft tissue contrast and is crucial in evaluating complex cases involving soft tissue tumours or malignancies. Additionally, CT is beneficial for identifying complications or differentiating between similar-appearing lesions, contributing to a comprehensive diagnostic approach. PET scans, while not routinely used for all umbilical lesions, play a crucial role in the staging and evaluation of metastatic diseases, including SMJNs, by highlighting areas of increased metabolic activity indicative of malignancy. The integration of these imaging modalities into clinical practice allows for a tailored and precise approach to diagnosing umbilical lesions, ensuring appropriate management strategies are implemented. Understanding the strengths and applications of each imaging technique enhances the ability to distinguish between benign and malignant conditions effectively, thereby improving patient outcomes. This comprehensive radiological assessment is essential for guiding surgical planning, monitoring treatment response, and providing prognostic information, underscoring the pivotal role of advanced imaging in the management of umbilical lesions.

## Conclusions

An umbilical lesion in adults that does not respond to typical treatment should raise the suspicion of a more sinister diagnosis that requires early detection and management to improve patient care and prognosis. The integration of clinical, radiological, and pathological data is essential in distinguishing malignant from benign nodules, facilitating early and effective cancer detection and treatment. This comprehensive approach not only aids in accurate diagnosis but also underscores the critical role of interdisciplinary collaboration in managing complex clinical presentations of umbilical nodules.
